# High Yielding Microbubble Production Method

**DOI:** 10.1155/2016/3572827

**Published:** 2016-02-29

**Authors:** Joe Fiabane, Paul Prentice, Ketan Pancholi

**Affiliations:** ^1^School of Engineering, The Robert Gordon University, Aberdeen AB10 7GE, UK; ^2^Institute for Medical Science and Technology, University of Dundee, Wilson House, 1 Wurzburg Loan, Dundee Medipark, Dundee DD2 1FD, UK

## Abstract

Microfluidic approaches to microbubble production are generally disadvantaged by low yield and high susceptibility to (micro)channel blockages. This paper presents an alternative method of producing microbubbles of 2.6 *μ*m mean diameter at concentrations in excess of 30 × 10^6^ mL^−1^. In this method, the nitrogen gas flowing inside the liquid jet is disintegrated into spray of microbubble when air surrounding this coflowing nitrogen gas-liquid jet passes through a 100 *μ*m orifice at high velocity. Resulting microbubble foam has the polydispersity index of 16%. Moreover, a ratio of mean microbubble diameter to channel width ratio was found to be less than 0.025, which substantially alleviates the occurrence of blockages during production.

## 1. Introduction

The generation of multilayered droplets, microbubbles, and double emulsions is fundamental to many applications such as drug delivery [1], cell therapy [2], material processing, environmental chemistry [3], and food [4]. Although many other methods such as liposomes [5], biocapsules [6], and nanoparticles [7] are available to deliver drugs to a target, the microbubbles are considered to be unique due to their capability of cell poration. The cell poration allows efficient delivery of drug to the cell. Drug delivery applications require a minimum of 1 million microbubbles of diameter between 1 and 10 *μ*m in 1 millilitre of liquid suspension [8]. Moreover, the formulation method should be capable of incorporating viscous drug within the microbubble structure. These demands pose an outstanding challenge for designing the method capable of generating the microbubble which meet the required limits on diameter and concentration.

Methods such as membrane emulsification, mechanical agitation, and laser or ultrasound induced cavitation are capable of generating small diameter microbubbles, but with a broad size distribution and do not facilitate simultaneous drug encapsulation [9]. However, the microfluidic based method allows control of the diameter, shell thickness, yield, and concentration of the microbubble [10, 11]. Additionally, this method also permits the encapsulation of fluid (drug), irrespective of its physical properties such as hydrophobicity and viscosity. Previous experimental and numerical studies have already demonstrated that injecting air into coflowing [12] or cross-flowing [13] liquid yielded the microbubbles with diameter less than the orifice diameter [14]. The additional stress imposed by the flowing liquid on to the injected air was found to be responsible for generating small microbubbles [15–18] and therefore, a simple control of the flow ratio *Q*_*g*_/*Q*_*l*_ can generate microbubble of required diameter. Here, *Q*_*g*_ and *Q*_*l*_ are the inner air and outer liquid flow rates, respectively [19]. If the similar microbubble production process is implemented in the two-dimensional microfluidic device [20], the diameter of the microbubbles is only controlled by the outer liquid flow, *Q*_*l*_. A substantial increase in *Q*_*l*_ causes change in flow regime from “dripping” to “jetting” [21]. In the dripping regime, the inner gas phase drips towards the orifice to ensure the generation of a monodisperse population of microbubbles, whilst a polydisperse population of microbubbles is generated in the jetting regime.

Alternatively, the diameter of the microbubble can also be controlled through controlling orifice diameter. The small orifice diameter can generate the microbubble with small diameter. However, the pressure required to force liquid and air through an orifice required large pumping pressure [22]. Furthermore, the option of increasing the diameter of the orifice has much appeal, as it would render a device less susceptible to blocking, a recurring problem in existing techniques [23]. However, the gas jet issuing from a large orifice has a larger cross-section, which in turn, generates larger microbubbles. As such, fine control over the flow instabilities in a larger capillary is required to produce smaller diameter microbubbles or droplets, with the capability of encapsulation [23]. This was achieved by means of a flow-focusing device with the crucial parameter of exit channel length : width ratio of 30. This high ratio combined with hydrophobic surface of the channel generates a strong pressure gradient in the focusing region and thus a long, narrow nitrogen gas thread forms, which is subsequently broken down into microbubbles. The break-up regime in such devices results in a highly monodisperse population of microbubbles (polydispersity index < 5%) [24]. Conversely, high velocity flow introduces velocity fluctuations at the interface of two immiscible phases, generating a polydisperse population of droplets (typical polydispersity index ~ 112%) [25].

In this work, a different approach of the microbubble break-up was taken. According to this new method, the nitrogen primary bubbles flowing inside the liquid jet were allowed to break up by the fast flowing air. The obtained results showed that this novel process could achieve a population of microbubbles with mean diameter, *d*_*s*_ much smaller than the exit orifice diameter, *d*_*c*_. Under optimal conditions, the new microbubbling device produced microbubbles of mean diameter 2.6 *μ*m and standard deviation of ±0.4 *μ*m with a yield in excess of 30 million per millilitre. In this process, three different fluids were passing through the orifice. The outermost phase was air while middle phase was liquid and innermost phase is nitrogen gas.

## 2. Device Design and Experimental Setup

**Figure 1:**
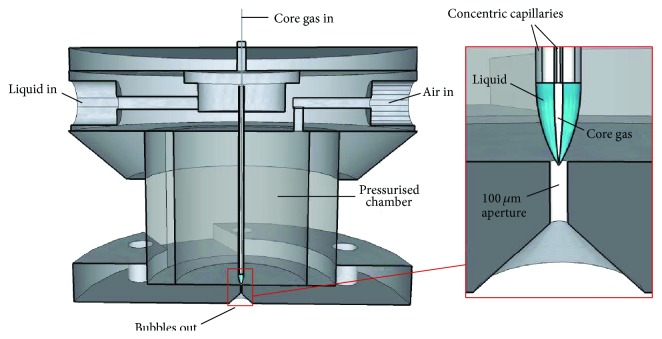
The microbubbling device (not to scale) consists of two concentric capillaries. The outer capillary carries the liquid whereas inner capillary carries core nitrogen gas. These coflowing liquid and gas phases are squeezed through the 100 *μ*m aperture by the surrounding air flow. On exiting the aperture, the core gas, in form of the primary microbubbles, is disintegrated into smaller secondary microbubbles.

The microbubbling device consisted of a pair of concentric capillaries exiting downwards into a chamber, which was pressurised with compressed air (Figure 1). The chamber was constructed from a cylindrical perspex tube with an internal diameter of 15 mm, secured between two plates via bolts arranged in a circle. The outer steel capillary was of internal diameter 584 *μ*m (outer diameter 890 *μ*m) and carried the liquid phase that form the shell material. The inner, silica capillary with internal diameter 50 *μ*m (outer diameter 360 *μ*m) supplied the core nitrogen gas. The steel and silica capillaries were secured to the top plate of the chamber using HPLC connections, capable of withstanding pressures of up to 48 MPa.

Additionally, the arrangement aligned both capillaries in a concentric fashion, while maintaining a distance of 0.6 mm from the tip of the steel capillary to a 100 *μ*m orifice in the bottom plate of the chamber, beyond which ambient atmospheric conditions exist. The air pressure inside the chamber, the nitrogen gas pressure, and liquid flow rate can be varied.

Schneider et al. [26], reported that the distance from the dispersed phase channel to the orifice (equivalent to the 0.6 mm gap here) is crucial to achieving a steady jet. Increasing this dimension beyond the optimum distance resulted in an increase in polydispersity index, indicative of jet instability, whilst a reduction resulted in larger bubbles being produced. This is most likely due to a reduction in the length of the nitrogen gas thread being subjected to axial pressure, and hence less “squeezing” of the thread before passing through the orifice. In our device, 0.6 mm was empirically found to produce microbubbles of the required size, while facilitating stable cone formation. The tip of the inner capillary was located just inside the steel capillary. A unique feature of this device was the ease with which capillaries can be replaced and steel capillary tip-to-orifice distance adjusted. Additionally, to the best of our knowledge, the diameter of the orifice and the capillary containing the liquid phase are significantly larger than most devices reported in the literature, capable of producing sub-10 *μ*m microbubbles [24]. This greatly facilitates the encapsulation of viscous liquids without the use of very high pressures. Similarly, clogging of the orifice (or other internal parts of the device) with the suspended shell material is considerably reduced. Increasing the orifice diameter beyond 100 *μ*m resulted in a smaller pressure drop across the aperture, causing formation of a larger diameter nitrogen gas thread, and thereby larger diameter microbubbles.

**Figure 2:**
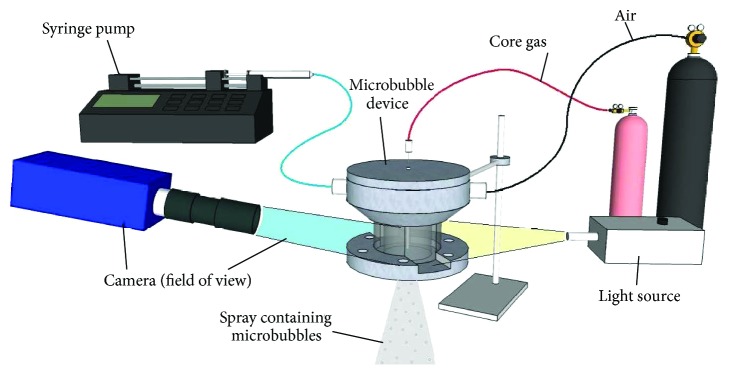
Experimental setup showing microfluidic connections supplying liquid, core nitrogen gas and air to the device. The camera was arranged to enable viewing of internal flows through the perspex wall of the chamber.

To prepare microbubbles [27], the device was connected to a syringe carrying the liquid phase, and pressurised gas canisters via flexible tubing, as indicated in Figure 2. A syringe mounted on a pump (Harvard PHD-4400, Harvard Apparatus Ltd., Edenbridge, UK) was supplying liquid at a constant flow rate, *Q*_*l*_, while the nitrogen gas bottle was supplying gas to the innermost capillary, at pressure *P*_*g*_. Compressed air was supplied at pressure *P*_*a*_, as described previously. During microbubble preparation, the air and nitrogen gas pressures were controlled by two-stage regulators and measured by a digital manometer. The device (Figure 2) was mounted in front of a camera (pco.1600, PCO AG, Kelheim, Germany) to capture real time images of liquid-nitrogen gas cone formation using a zoom lens (Navitar, Inc., Rochester, NY, USA), recorded to a PC for further analysis.

## 3. Materials and Methods

### 3.1. Lipid Suspension Preparation

The liquid used to prepared microbubbles was a lipid suspension. Specifically, phospholipid (1,2-distearoyl-sn-glycero-3-phosphocholine (DSPC), Avanti Polar Lipids, Alabaster, AL, USA) and surfactant (PEG-40-stearate, Sigma, St. Louis, MO, USA) were mixed in a ratio of 9 : 1 mol/mol, and dissolved in the chloroform. The chloroform was evaporated using a rotary evaporator (Büchi, Flawil, Switzerland) to form a thin film. Enough care was taken in setting temperature and vacuum pressure so that the chloroform can be evaporated without changing lipid phase. The residual chloroform was not measured but a lipid film was left under nitrogen flow for enough time so as to remove any chloroform residue. The film was resuspended in 90 : 10% distilled water : glycerol solution to achieve 3 mg/mL lipid concentration and sonicated using a bath-type sonicator (Branson Ultrasonics Corp., Danbury, CT, USA) for two hours at 37°C, to disperse the solids completely. The resulting suspension was stirred overnight to ensure air saturation. In further experiments, other hydrophilic stabilisers, PEG polymers with molecular weights of 1500 and 4000 Daltons (Sigma, St. Louis, MO, USA) were used in place of PEG-40-stearate to enhance viscosity and stability of the microbubbles [28, 29]. Two different concentrations of each stabiliser (0.5 and 1%) were prepared to determine their effects on stability.

### 3.2. Microbubble Preparation and Analysis

To observe the effect of various parameters on microbubble diameter and size distribution, the core nitrogen gas pressure, *P*_*g*_, outer air pressure, *P*_*a*_, and liquid flow rate, *Q*_*l*_, were varied to form a range of parametric combinations. These combinations were chosen to measure the combined effect of each parameter in relation to others. In this study, liquid flow rate *Q*_*l*_ was varied from 1.6 × 10^−9^ m^3^/s to 1.6 × 10^−8^ m^3^/s, the air pressure *P*_*a*_ was varied from 80 to 800 kPa, and nitrogen core gas *P*_*g*_ was varied from 80 to 750 kPa.

**Figure 3:**
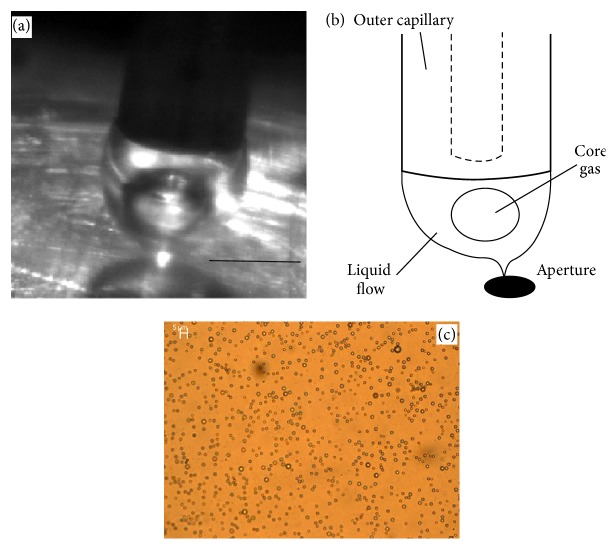
(a) Selected image shows nitrogen core primary bubble forming inside liquid cone. The primary nitrogen gas bubbles issuing from the inner-most capillary were subsequently fragmented into secondary microbubbles while passing through the aperture. Scale bar represents 60 *μ*m. (b) The schematic shows the same process as captured in (a). However, it includes label. (c) A micrograph of the secondary microbubble population produced under optimal conditions. The micrograph of the microbubbles illuminated with white light was captured using Leica DC150 at 50x magnification and the scale bar on the micrograph represents 5 *μ*m.

The desired microbubble structure consists of a gas core, surrounded by a shell of phospholipid and surfactant, suspended in the water [30]. The first step of the operation was to begin the flow of the core nitrogen gas, at a relatively low pressure initially (typically 20 kPa), to prevent the liquid phase entering back into the core nitrogen gas tubing, when the air pressure was increased. The flow of the liquid phase was then initiated using the syringe pump. As the fluids emerged from the capillaries, they form a two-phase flow with large “primary” bubble of diameter *d*_*p*_ emerging from the inner capillary into the liquid, which subsequently was fragmented into “secondary” microbubbles, of mean diameter, *d*_*s*_ as they pass through the orifice (Figure 3(c)).

The outer air pressure was then increased gradually until the two-phase flow resembled a cone, and a fine jet and mist was emitted from an orifice. At the same time, the core nitrogen gas pressure must be gradually increased to maintain its flow, counteracting the air pressure in the chamber. Flow of the core nitrogen gas can be confirmed visually using the high speed video on the PC, to observe the formation of primary bubbles within the cone. Typically the core nitrogen gas pressure must be at least 100 to 150 kPa to maintain flow for a chamber pressure of 200 kPa. The flow of air exiting the device drew the two-phase (nitrogen gas inside liquid) flow towards the orifice forming a cone shape (Figures 3(a) and 3(b)). The air passing through the orifice, alongside the two-phase flow, exerted pressure normal to the two-phase compound jet. This reduced it into a very fine thread as it exited the device, whereupon it broke down into a fine spray containing the microbubbles. This typically occurred at pressures of around *P*_*a*_ = 250 kPa and *P*_*g*_ = 150 kPa, for a liquid flow rate of *Q*_*l*_ = 6.4 × 10^−9^ m^3^/s.

**Figure 4:**
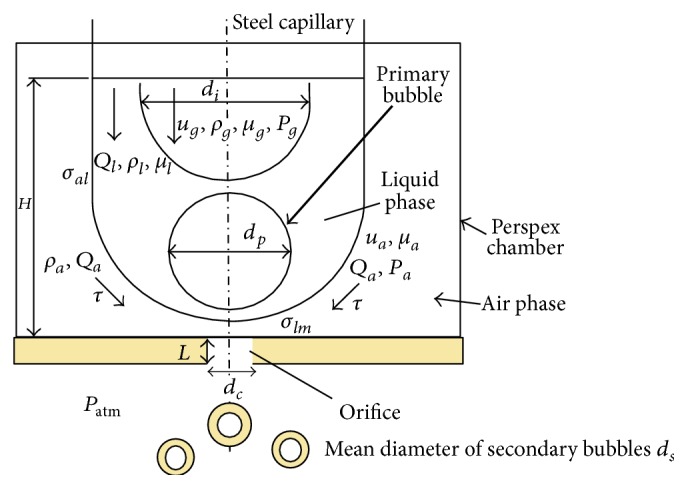
Schematic showing nitrogen gas propagating inside the liquid jet in vicinity of the orifice. The nitrogen gas breaks up into primary bubbles and is further broken down into secondary bubbles as it flows through the orifice along with surrounding air phase. All associated parameters and its notations are labelled.

The bubble formation process was (Figure 4) recorded with a high speed camera (resolution 512 × 512 pixels) at a temporal resolution of 29 *μ*s per frame and Camware software version 2 (PCO AG, Kelheim, Germany). Throughout an experiment, the camera field view was set at approximately 10 mm × 7.5 mm and for each parametric combination, at least 150 images of stable liquid-nitrogen gas cone were acquired. All images were calibrated using the steel capillary outer diameter as a known reference dimension, from which a scale of 1 *μ*m per pixel is deduced. To analyse nitrogen gas propagation velocity, a curve was fitted to the nitrogen gas-liquid interface for the primary bubble inside the liquid cone [31]. Tracking the movement of the point of maximum curvature allowed the nitrogen gas propagation velocity to be measured. All analysis procedures including measurement of various spatial dimensions were accomplished using ImageJ software (US National Institutes of Health, Bethesda, MD, USA).

In this way, the velocity of the core nitrogen gas was measured for different liquid flow rates from 1.6 × 10^−9^ to 1.6 × 10^−8^ m^3^/s. The experimentally measured velocity was theoretically validated employing a geometrical approach [31], whereby the nitrogen gas propagation inside the two-phase flow was assumed to be a combination of radial and linear components.

This system is capable of producing microbubbles down to approximately 1/40th of the actual orifice size (Figure 3(b)), at low air pressures of circa 250 kPa.

### 3.3. Size Distribution Studies

The secondary microbubbles emerging from the orifice were collected on a slide for immediate microscopic examination or stored in an ice cooled vial (4°C) for size distribution analysis. Temporal changes in the microbubble population in each of three samples having different stabiliser compositions (PEG-2000, PEG-4000, and PEG 40S) were analysed for stability after 1 hour using an upright microscope (Nikon, Japan) and a hemocytometer slide. Images captured at 20x or 40x magnifications were processed in Image Pro Plus software (Media Cybernetics, Bethesda, MD, USA) using size/count function to obtain nitrogen microbubble counts and mean diameters at respective times. Analysis indicated maximum stability for the secondary microbubble suspension with PEG-40 stearate stabiliser; hence this was chosen for all subsequent studies.

## 4. Results and Discussion

### 4.1. Experimental Observations

The air flow Reynolds number Re_*a*_ = *Q*_*a*_ *ρ*_*a*_/*μ*_*a*_ *d*_*c*_ in the vicinity of the orifice was in the range of 1100 to 4500 for range of *Q*_*a*_. The schematic of the process of bubble break-up can be represented as shown in Figure 4. Hereafter *Q*, *u*, *ρ*, *μ*, *σ*, and *P* will be used to denote flow rate, velocity, density, viscosity, interfacial tension, and pressure, respectively, whereas subscripts *a*, *al*, *lg*, *lm*, and *l* will indicate air, liquid-air, nitrogen gas-liquid, liquid-orifice plate, or liquid (Figure 4).

In this process, a microbubble population with a narrow size distribution (2.6 *μ*m ± 0.4 *μ*m) was produced, despite the high Re_*a*_. A high Re_*a*_ air flow must have generated nonlinear velocity fluctuations at the liquid-air interface of the two-phase compound jet. Such fluctuations generally lead to the generation of highly polydisperse microbubble populations (polydispersity index > 112%) [25]. In this work, however, the high Re_*a*_ air flow surrounding the compound jet produced a microbubble population with polydispersity index of 16%. The behaviour of the fluids in the device and the resulting microbubble populations are analysed as discussed previously.

The observations of the microbubble formation process are categorised into three distinct regimes.

### 4.2. Low Air Pressure Regime

**Figure 5:**
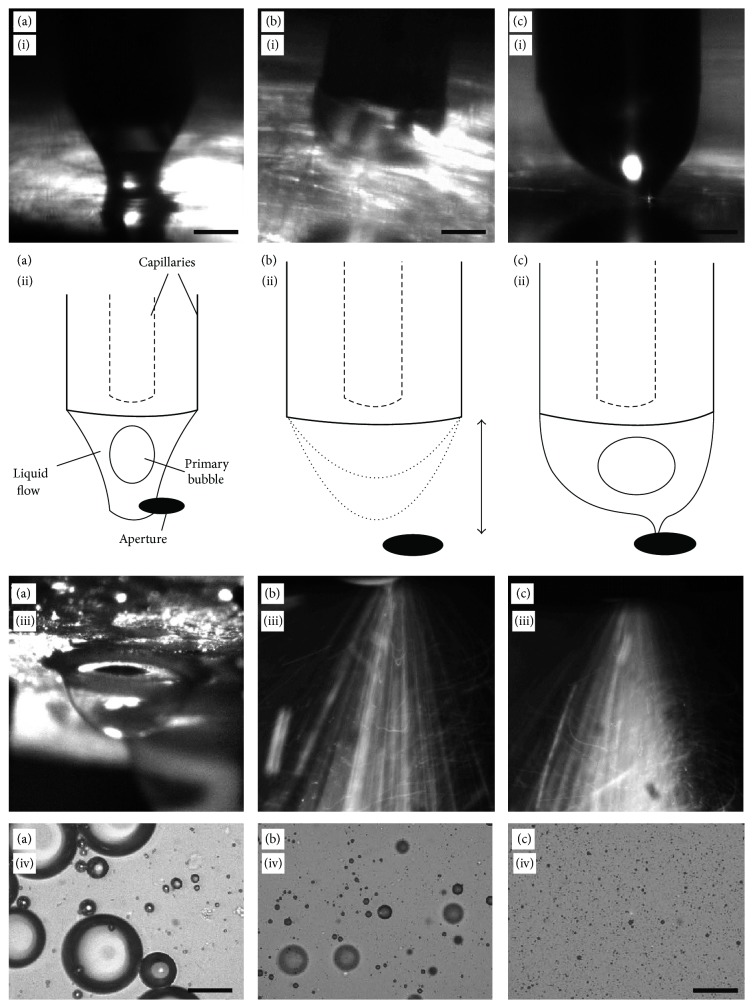
The nitrogen gas-liquid compound jet behaviour in the different flow regimes and the resulting microbubble population. (a) (i, ii) Due to insufficient air pressure, the liquid-nitrogen gas cone contacts the bottom surface of the chamber before flowing out through the orifice. (iii) The large bubbles grow and burst outside the orifice, (iv) to produce large diameter bubbles. (b) (i, ii) In intermediate air regime, the compound jet drips from the inner capillary and emits through orifice (iii) as intermittent sprays. Resulting microbubble population is shown in (iv). (c) (i, ii) At the optimised pressure regime, a stable gas-liquid cone is disintegrated into (iii) fine spray-mist outside the orifice. (iv) The microbubbles with 2.6 *μ*m mean diameter and polydispersity index of 16% were produced. Scale bar for all images represents 50 *μ*m.

Firstly, (Figure 5(a)(i–iv)), the values of *P*_*g*_ and *P*_*a*_ were set low (typically *Q*_*l*_ = 6.4 × 10^−9^ m^3^/s, *P*_*g*_ = 80 kPa, and *P*_*a*_ = 100 kPa) for a constant value of *Q*_*l*_, until the liquid was seen emerging from the steel capillary. Since the pressure ratio across the orifice was small, a cone was not formed, and the liquid dripped from the steel capillary (Figure 5(a)(i), (ii)) onto the bottom surface of the chamber to block the orifice. Since air passage through orifice was blocked, the *P*_*a*_ values kept increasing until it forced the liquid out through the orifice causing large (~0.3 mm) nitrogen gas bubbles to burst through (Figure 5(a)(iii)), ejecting droplets of liquid and microbubbles erratically in all directions. After some time, the primary nitrogen bubble started forming within the wide cone. These primary bubbles passed through the orifice forming nitrogen gas-in-liquid compound jet inside the orifice. However, the lower air pressure around the compound jet was causing random and slower break-up. The resulting microbubble populations contained high numbers of larger microbubbles and a small number of finer microbubbles. Some microbubbles with diameters were found to be as large as 50 micrometres (Figure 5(a)(iv)).

### 4.3. Medium Air Pressure Regime

In the second regime (Figure 5(b)(i–iv)), an increase in the values of *P*_*g*_ and *P*_*a*_ (typically *P*_*g*_ = 120 kPa, *P*_*a*_ = 200 kPa) resulted in the reduction in erratic behaviour as the liquid did not come into contact with the orifice. Moreover, an increase in pressure of the air increased the frequency of the dripping but the dripping did not stop completely. As each drip first emerged from the steel capillary, the nitrogen gas was seen entering the liquid phase from the inner capillary. The drip then travelled downwards (Figure 5(b)(i)) until it was close enough to the orifice for the surrounding air flow to draw the liquid through. The drip containing liquid-air interface then retreated to the capillaries before the process repeated again. This entire process appeared as intermittent sprays exiting from the orifice (Figure 5(b)(iii)) at the “drip” frequency (typically 10 Hz, although dependent on liquid flow rate and air pressure) and was accompanied by characteristic pulsed spraying sound. Since air velocity was not high enough, the air-flow induced inertial force may be just enough to overcome the surface tension of the compound jet and therefore, it did not stabilise the jet. The resulting instability might have promoted irregular break-up of the jet generating more numbers of smaller and fewer numbers of larger microbubbles [32]. The resulting product contained a population with significantly tighter size distribution, although still polydisperse, with a small number of larger microbubbles, circa 15–20 *μ*m in diameter (Figure 5(b)(iv)).

### 4.4. High Air Pressure Regime

As the values of *P*_*a*_ and *P*_*g*_ were increased further, the microbubble production entered the final regime as shown in Figure 5(c)(i–iv), (typically *P*_*g*_ = 150 kPa, *P*_*a*_ = 250 kPa). During the final regime, the dripping behaviour in the chamber ceased and the compound jet remained adjacent to the orifice. As shown in Figure 5(c)(i), the liquid cone containing series of primary nitrogen gas bubbles squeezed into a continuous fine thread before passing through the orifice without contacting the side walls of the orifice. The continuous moving stream of the primary nitrogen gas bubbles was visible on the high speed camera images. Outside the orifice, a fine spray was emitted, without the pulsatile behaviour that characterised regime II (Figure 5(c)(iii)). In this final regime, the high air pressure increased the air velocity around the compound jet while passing through the orifice, which must have increased inertial force and resulted into the finer spray due to short wavelength pressure transients [31]. This parametric combination yielded the microbubble population with much smaller mean diameter and polydispersity index of 16%.

#### 4.4.1. Size Distribution and Stability

After an hour, the nitrogen microbubble population in ten samples was found to be reduced to 16 ± 2% of its original population of 3 × 10^7^ mL^−1^. The nitrogen gas diffused into surrounding liquid much faster than the large molecule gas such as perfluorobutane (PFB) and therefore, stability of the microbubble with nitrogen gas core was found to be less than an hour in our experiments. The stability for the microbubbles with PEG 1500 and 4000 was found to be very less and therefore, it is not reported here. These two molecules worked as a stabiliser to reduce the surface tension, which was not enough to stabilise the small diameter microbubbles for a long time. However, the amphiphilic PEG 40-S was found to stabilise the microbubbles (~2.4 *μ*m diameter) for approximately an hour as these molecules disperse the lipid and allow self-assembly of the amphiphilic molecules at the air-liquid interface. The self-assembly reduces surface tension at the interface and inhibits the gas dissolution to increase stability of the microbubbles. With the use of PFB gas, the stability is expected to increase manifold for microbubbles of diameter in range of 2.4 *μ*m.

**Figure 6:**
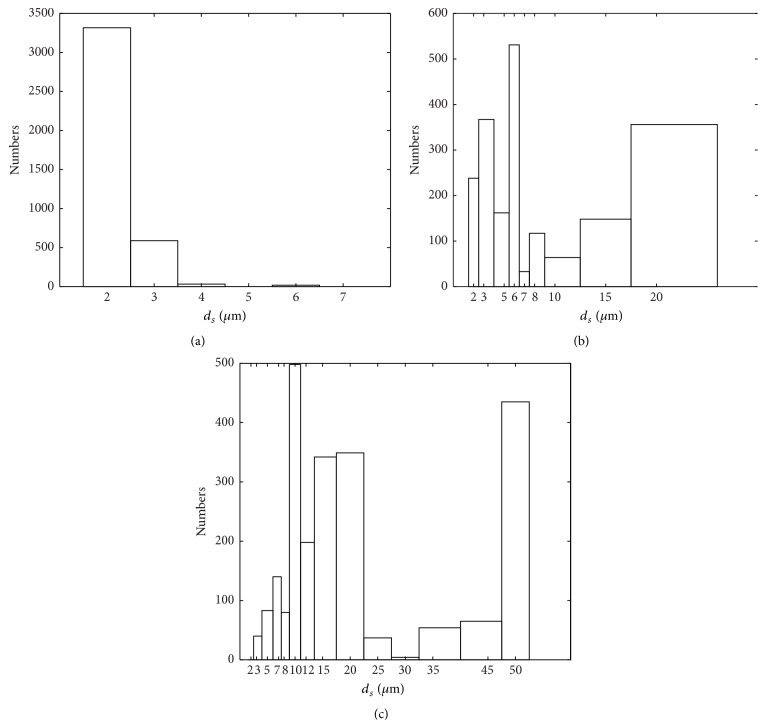
Representative size distribution plots of nitrogen encapsulated microbubbles recorded at different pressure ratio. (a) *P*_*g*_/*P*_*a*_ ≤ 0.6, (b) 0.6 ≤ *P*_*g*_/*P*_*a*_ ≤ 0.8, and (c) 0.8 ≤ *P*_*g*_/*P*_*a*_ ≤ 1.1.

Figure 6(a) shows the microbubble size distribution when pressure ratio was less than or equal to 0.6 (*P*_*g*_/*P*_*a*_ ≤ 0.6) and this refers to the third regime of the bubble break-up. For smaller pressure ratio, the gas pressure was far lower than the air pressure which must have efficiently fragmented the primary bubbles into smaller microbubbles. The microbubble size distribution for the pressure ratios between 0.6 ≤ *P*_*g*_/*P*_*a*_ ≤ 0.8 is skewed towards larger diameters (Figure 6(b)), which shows that the less number of primary bubbles must have undergone the break-up. However, in the higher range, 0.8 ≤ *P*_*g*_/*P*_*a*_ ≤ 1.1, most of primary bubbles may have not gone through break-up as the large quantity of microbubbles with diameter in range of 50 and 10 *μ*m were found in the sample (Figure 6(c)). If the velocity difference between the inner nitrogen gas phase and outer air is above its critical value, the probability of encapsulating inner nitrogen gas reduces [33]. This has also been observed experimentally elsewhere [34].

It was observed that the high liquid flow rate did not significantly affect the mean diameter of the microbubbles [35]. However, it was a precondition for obtaining a cone and subsequent encapsulation. At very low liquid flow rate, the inner nitrogen gas cannot be confined within the liquid, resulting in unsuccessful encapsulation as well. If the liquid flow is increased beyond a certain value in relation to the gas and air velocities, it may choke the orifice or flood the chamber.

## 5. Conclusions

The device capable of generating microbubbles with mean diameter as low as 2.6 *μ*m and standard deviation of ±0.4 *μ*m is reported here. The produced microbubbles were as small as one-fortieth of the orifice and their mean diameter can be controlled by varying liquid flow rate and nitrogen gas pressures. Unlike the randomness observed in the break-up of droplets or bubbles in the higher Reynolds number regime, in this work the break-up of the primary nitrogen gas bubbles in high air Reynolds number regime did not result in the high polydispersity of the resulting secondary microbubbles.
